# A Comparison of Sms 201-995 and Oesophageal Tamponade in the Control of Acute Variceal Haemorrhage

**DOI:** 10.1155/1992/76345

**Published:** 1992

**Authors:** Ruth F. Mckee, O. J. Garden, J. R. Anderson, D. C. Carter

**Affiliations:** 1 University Department of Surgery Royal Infirmary Glasgow United Kingdom; 2 Ward 49/50 Aberdeen Royal Infirmary Foresterhill Aberdeen AB9 2ZB UK

## Abstract

Forty endoscopically proven active variceal bleeds were entered in a prospective trial comparing oesophageal tamponade with SMS 201-995 infusion.

Oesophageal tamponade controlled 19 of 20 bleeds over the first four hours and 14 of 18 bleeds over 48 hours. SMS 201-995 infusion controlled 18 of 20 bleeds over the first four hours and 10 of 20 bleeds over 48 hours (*p* = 0.15). No significant differences between the groups were seen in time to control of bleeding, amount of blood transfused or number of patients crossed over to the opposite treatment. Complications in the oesophageal tamponade group were discomfort due to the tube (17 patients) and chest infection (10 patients), while in the SMS 201-995 group 7 chest infections and one episode of hyperglycaemia occurred, with no symptomatic complaints. The patient survived the admission in 15 of the oesophageal tamponade bleeds and all of the SMS 201-995 bleeds (*p* = 0.047).

An intravenous infusion of SMS 201-995 appears to have comparable efficacy to oesophageal tamponade in variceal bleeding.


HPB Surgery, 1992, Vol. 6, pp. 7–17
Reprints available directly from the publisher
Photocopying permitted by license only

© 1992 Harwood Academic Publishers GmbH
Printed in the United Kingdom

### A COMPARISON OF SMS 201-995 AND OESOPHAGEAL TAMPONADE IN THE CONTROL OF ACUTE VARICEAL HAEMORRHAGE

RUTH F. McKEE, O.J. GARDEN, J.R. ANDERSON and D.C. CARTER
*University Department of Surgery, Royal Infirmary, Glasgow, United Kingdom*

*(Received 27 December 1991)*

Forty endoscopically proven active variceal bleeds were entered in a prospective trial comparing oesophageal tamponade with SMS 201-995 infusion.

Oesophageal tamponade controlled 19 of 20 bleeds over the first four hours and 14 of 18 bleeds over 48 hours. SMS 201-995 infusion controlled 18 of 20 bleeds over the first four hours and 10 of 20 bleeds over 48 hours ($p = 0.15$). No significant differences between the groups were seen in time to control of bleeding, amount of blood transfused or number of patients crossed over to the opposite treatment.

Complications in the oesophageal tamponade group were discomfort due to the tube (17 patients) and chest infection (10 patients), while in the SMS 201-995 group 7 chest infections and one episode of hyperglycaemia occurred, with no symptomatic complaints. The patient survived the admission in 15 of the oesophageal tamponade bleeds and all of the SMS 201-995 bleeds ($p = 0.047$).

An intravenous infusion of SMS 201-995 appears to have comparable efficacy to oesophageal tamponade in variceal bleeding.

KEY WORDS: Somatostatin analogue, variceal haemorrhage

### INTRODUCTION

There is considerable debate as to the best means of safely establishing immediate control of acute bleeding from oesophageal varices. Oesophageal tamponade has been shown to be an effective means of control of bleeding[1,2,3] but respiratory complications may arise and patient tolerance is poor. Vasopressin has been the mainstay of drug treatment over the past thirty years but doubts have been raised as to its efficacy[4] and serious cardiac side-effects may occur. It has been suggested that the addition to vasopressin of a vasodilator such as nitroglycerin may reduce the incidence of side-effects[5,6] but the use of a single drug without generalised cardiovascular side-effects would be simpler. Emergency injection sclerotherapy has the advantage of initiating definitive treatment rather than only providing temporary arrest of bleeding[7]. However considerable expertise is required to perform sclerotherapy in an actively bleeding patient and prior control of bleeding may still be needed to permit adequate resuscitation before sclerotherapy can be undertaken.

We have previously established that a new, long-acting somatostatin analogue,

Address correspondence to: Dr Ruth F. McKee, Ward 49/50, Aberdeen Royal Infirmary, Foresterhill, Aberdeen AB9 2ZB, UK

SMS 201-995 (Sandostatin, Sandoz Ltd, Basel), causes a 30% reduction in portal pressure in stable cirrhotic patients[8]. In two clinical trials, naturally occurring somatostatin has been shown to be at least as effective as vasopressin in controlling variceal haemorrhage, with fewer side-effects[9,10]. However practical problems arise during its administration due to its instability in solution and its very short half-life. Furthermore, rebound phenomena have been observed on completion of intravenous infusions of somatostatin[11]. No rebound phenomena have been observed after infusion of SMS 201-995, a long-acting octapeptide with a half-life of 45 minutes in plasma, and its effect on insulin levels is small and short lasting in comparison to its other effects[12]. In the present study we have compared the efficacy of SMS 201-995 with the Minnesota modification of the Sengstaken Blakemore tube in early control of active variceal bleeding.

### PATIENTS AND METHODS

Between July 1986 and May 1988, patients admitted to the University Department of Surgery, Glasgow Royal Infirmary who were demonstrated to have active bleeding from oesophageal varices at endoscopy were considered for the study. Portal hypertensive patients with bleeding from other sources were excluded as were patients who had experienced a myocardial infarction within the preceding six months, patients with renal failure requiring dialysis and patients with insulin dependent diabetes mellitus. No patient was on prophylactic beta blockade to prevent variceal rebleeding.

Following admission to hospital patients were resuscitated with blood and plasma and fibreoptic endoscopy under sedation (intravenous diazemuls 0 to 30 mg) was performed within two hours. Prior to endoscopy in patients suspected of having variceal haemorrhage, the trial was explained and verbal consent obtained from either the patient or a relative. Having confirmed active bleeding from oesophageal varices at endoscopy patients were randomised to treatment with SMS 201-995 or oesophageal tamponade using numbered sealed envelopes. The amount of blood transfused prior to endoscopy and the time from the start of overt bleeding were recorded. Patients treated with SMS 201-995 were given an intravenous infusion of 25$\mu$g/hr in 120ml 0.9% saline over 48 hours using a syringe pump (Treonic IP4, Vickers Medical). Patients randomised to oesophageal tamponade had the Minnesota modification of the Segstaken Blakemore tube (4 lumen) passed through the mouth or nose by one of three experienced members of medical staff[2]. The gastric balloon was inflated with 100ml of saline and 20ml of water-soluble contrast medium. The oesophageal balloon was inflated to 40mm Hg as indicated by an anaeroid barometer and the tube taped to the cheek under slight tension. The tube position was confirmed by chest X-ray immediately after insertion and the patient was constantly supervised by a trained nurse who aspirated the gastric and phparyngeal lumina of the tube hourly. Between aspirations the lumina remained on open drainage. The tube was left in place for 24 hours with both balloons inflated. At 24 hours the oesophageal balloon was deflated and the tube untaped so that the gastric balloon lay free in the stomach. This has been our standard method of emergency treatment for variceal haemorrhage for some years, with ability to control bleeding in 94% of cases in the past[2] and similar methods are used by other groups[1,3]. If further bleeding occurred the oesophageal balloon was re-inflated and the tube taped as before. The tube was removed at 48 hours.

Whole blood and plasma were administered for volume and red cell replacement. Vitamin K, fresh frozen plasma, platelets and/or cryoprecipitate were given as appropriate if a coagulation defect was demonstrated at the time of the bleed. Oral lactulose and regular saline rectal washouts were used to prevent or treat encephalopathy. All patients were closely monitored by hourly blood pressure, pulse, urine volume and temperature measurements. Patients given SMS 201-995 infusion had a nasogastric tube passed to monitor gastric aspirations hourly. At the end of the trial period all patients underwent a further fibreoptic endoscopy to assess control of bleeding and to perform injection sclerotherapy. This study was approved by the Ethical Committee of the Royal Infirmary, Glasgow.

All patients had clinical examination and the following investigations performed at 0 and 48 hours: full blood count, coagulation screen, serum urea and electrolytes, blood glucose, liver function tests, plasma proteins and arterial blood gases. An electrocardiogram and chest X-ray were performed at the beginning and the end of the 48 hour period. Pugh's modification of Child's grading[13] was used as an assessment of liver function and was recorded at 0 and 48 hours for each patient. In addition, full blood count and blood glucose were repeated 12 hourly and urea and electrolytes and liver function tests repeated at 24 hours. Two successive creatinine clearance measurements were also performed during the 48 hour study period. Clinical examination, chest X-ray and arterial blood gases were repeated at 72 hours and 7 days from admission to the trial. Fluid balance and requirements for blood and blood products were recorded.

Cessation of bleeding was recorded at the hour when less than 20ml of fresh blood was aspirated from the stomach provided there was no other evidence of bleeding. Rebleeding was deemed to have occurred if one or more of the following criteria were fulfilled:

(a) overt haemorrhage or aspiration of more than 100ml of fresh blood
(b) passage of fresh blood per rectum
(c) fall in haemoglobin concentration of more than 4g/dl within 48 hours
(d) shock (pulse rate $>100$; systolic blood pressure $<100$) in the presence of continuing melaena.

If either treatment failed to control the bleeding or if rebleeding occurred during the 48 hours of treatment the patient was crossed over immediately to the other treatment. Control of bleeding was assessed during the first four hours of the trial period (recorded as initial control), during the first 24 hours and for the whole 48 hour period (complete control). The further progress of the patient during that admission to hospital was recorded, with particular reference to survival during that admission and cause of death. Symptomatic side-effects were assessed by enquiry from the patient. Evidence of chest infection was assessed by clinical examination, serial chest X-rays and blood gas analysis and was deemed to be present if CXR showed collapse or consolidation, pO2 deteriorated by more than 15 mm Hg and/or clinical examination revealed coarse crepitations with the production of purulent sputum.

Comparisons of the two groups with regard to time to bleeding control from start of treatment, amount of blood transfused and creatinine clearance values were carried out using the students *t*-test. Comparison of the control rate at 24 and 48 hours were performed using Chi-squared tests. Bleeding control rate at 4 hours, number of rebleeds, number of crossovers and survival were compared using Fisher's exact test. $p < 0.05$ was taken as the level of statistical significance.

### RESULTS

The results of this trial are summarised in Figure 1. During the 23 month trial period, 96 episodes of variceal bleeding occurred in 72 patients. Forty separate admissions with endoscopically proven active variceal bleeding were included. Fifty-six bleeds in 41 patients were excluded (Table 1). Thirteen of these excluded bleeds proved fatal, either due to the bleed itself or its complications.

Forty episodes of variceal haemorrhage in 31 patients were included in the trial. Twenty bleeds were randomised to oesophageal tamponade (OT) and 20 bleeds to SMS 201-995 infusion (SMS). These two groups were comparable with regard to age, sex distribution and aetiology of portal hypertension (Table 2).

**Table 1** The reasons for exclusion from the trial of 56 other bleeds thought to be from oesophageal varices

| | |
|---|---|
| No active bleeding after admission | 31 |
| Transfer from another hospital with Minnesota tube in place | 11 |
| Danger of exsanguination-tamponade instituted before endoscopy possible | 5 |
| Moribund on admission and could not be resuscitated | 2 |
| Wrong initial diagnosis of Mallory-Weiss tear | 1 |
| No active treatment–terminal disease | 4 |
| Emergency injection sclerotherapy in error | 1 |
| Excluded in error because of age | 1 |
| Total | 56 |

**Table 2** Patient characteristics for the two groups treated with oesophageal tamponade or SMS 201-995 infusion for active variceal bleeding

| | *Oesophageal Tamponade* | *SMS 201–995 Infusion* |
|---|---|---|
| Total number | 20 | 20 |
| Sex Ratio (M:F) | 16:4 | 18:2 |
| Age in years – mean (sem) | 51.6 (3.3) | 49.9 (3.5) |
| Aetiology | | |
| Alcoholic cirrhosis | 13 | 15 |
| Primary biliary cirrhosis | 1 | 1 |
| Chronic active hepatitis | 3 | 0 |
| Cryptogenic cirrhosis | 3 | 1 |
| Sclerosing cholangitis | 0 | 1 |
| Drug induc ed cirrhosis | 0 | 1 |
| Congenital hepatic fibrosis | 0 | 1 |
| Pugh's modification of Child's grade | | |
| A | 1 | 2 |
| B | 4 | 8 |
| C | 15 | 10 |

The characteristics of the variceal bleeds included in this trial in terms of the number of first bleeds from varices, the time from its initial manifestation and the amount of blood given before admission to the trial were similar in the two groups (Table 3).

**Table 3** Characteristics of the bleed for the two groups treated with oesophageal tamponade or SMS 201–995 infusion for active variceal bleeding

| | *Oesophageal Tamponade* | *SMS 201–995 Infusion* |
|---|---|---|
| Number of first bleeds | 6 | 9 |
| Hours from start of overt bleeding – mean (sem) | 20.5 (4.2) | 17.9 (3.6) |
| Number of patients transfused before admission to trial | 11 | 9 |
| Mls of blood transfused per patient transferred before admission to trial – mean (sem) | 1464 (308) | 1867 (291) |

### *Control of Bleeding (Figure 1)*

Bleeding was controlled during the first 4 hours after admission to the trial in 19 of 20 bleeds treated with oesophageal tamponade and 18 of 20 bleeds treated with SMS 201-995 infusion. In the one bleed in which haemorrhage was not controlled by tamponade, crossover to SMS 201-995 infusion was undertaken at two hours but this failed to control the bleeding. Bleeding was controlled by injection sclerotherapy but the patient died of hepatorenal failure at 40 hours from admission to the trial. This patient had a very poor prognosis on admission, having Grade III encephalopathy and a prothrombin time of 40 seconds[13]. In the two instances where haemorrhage was not controlled by SMS 201-995 infusion during the first 4 hours, crossover to oesophageal tamponade resulted in cessation of the bleeding.

In the oesophageal tamponade group 15 bleeds stopped immediately treatment was started, while in four other patients small amounts of fresh blood (<100ml/hr, stopped by 4 hours) were aspirated for several hours before bleeding stopped completely (Figure 1). In the SMS 201-995 group 11 bleeds stopped completely within the first hour while seven bleeds took longer to settle. This difference in time to control bleeding is not statistically significant ($p = 0.24$).

Over the 48 hour trial period, two patients in the oesophageal tamponade group died, the patient mentioned above whose bleeding was uncontrolled by tamponade and SMS 201-995 infusion, and one patient who died at 4.5 hours from admission due to severe metabolic upset secondary to preexisting hepatorenal failure. At 24 hours from admission, 16 of 19 patients alive in the OT group had no further bleeding. In two patients rebleeding occurred during the first 24 hours when the oesophageal balloon should have been inflated, but in one instance this had been accidentally deflated and in the other the oesophageal balloon had burst. In 14 of the remaining 18 bleeds in the OT group there was no further bleeding over 48 hours. Two other rebleeds occurred during the second 24 hours when the oesophageal balloon was deflated. In the OT group all four of these rebleeds were easily controlled by either re-inflation of the oesophageal balloon or the insertion of a

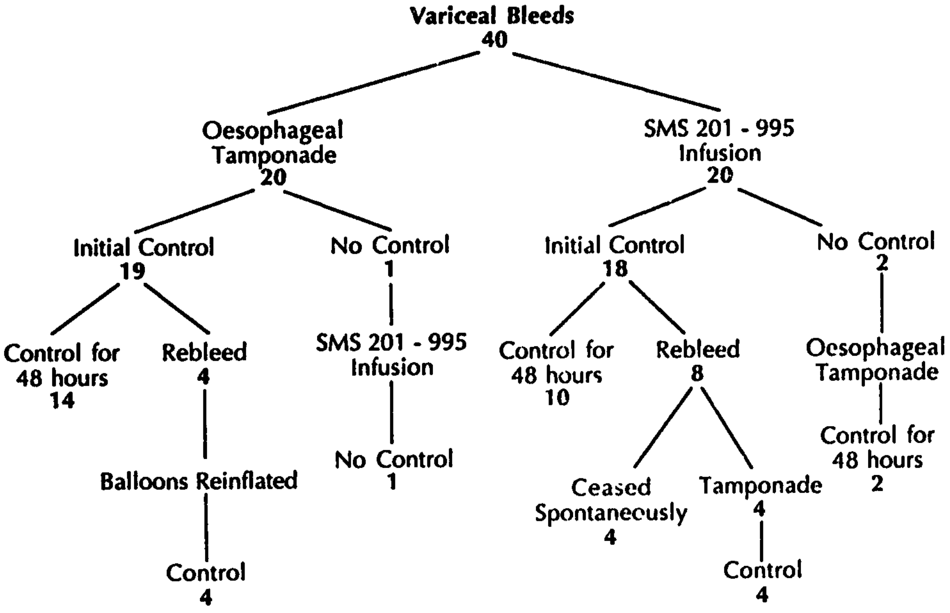

**Figure 1** The clinical course in 40 active variceal bleeds entered in this study of oesophageal tamponade compared to SMS 201-995 infusion, with particular regard to control of bleeding.

new Minnesota tube in the case where the balloon had burst. Therefore no patient required crossover to SMS 201-995 infusion because of rebleeding.

In the SMS group 14 of 20 patients had no further bleeding at 24 hours ($p < 0.5$ cf. OT group) and 10 patients had had no bleeding at 48 hours. In 8 instances there was evidence of rebleeding but in four cases this settled without treatment. Four rebleeds required crossover to oesophageal tamponade and the bleeding was controlled by this in each case. The difference in 48 hour bleeding control rate is not statistically significant ($p = 0.15$).

The 20 bleeds in the oesophageal tamponade group required a mean of 1680 mls (sem 295 mls) of blood transfusion over the 7 day trial period while the 20 bleeds in the SMS 201-995 group required a mean of 1710 mls (sem 252 mls) ($p = 0.4$).

*Complications*

In 22 of the 26 bleeds treated at some time by oesophageal tamponade, the patient complained of discomfort caused by the Minnesota tube. All of the other 4 patients had hepatic encephalopathy. In the 21 bleeds treated with SMS 201-995 infusion there were no symptomatic side-effects.

No patient in the OT group required insulin therapy to control hyperglycaemia. In the SMS group, one patient who was a no insulin dependent diabetic required an intravenous infusion of soluble insulin of 1–2 units/hour to maintain his plasma glucose below 15 mmol/l but this easily controlled the plasma glucose level which remained stable when the SMS 201-995 infusion was stopped. No other patient required insulin infusion despite the fact that two other bleeds in the SMS group occurred in maturity onset diabetics.

In 15 of the bleeds treated with oesophageal tamponade two consecutive creatinine clearance measurements were made. The mean of the mean creatinine clearance values for each individual was 106ml/min (sem 10). The mean creatinine clearance rate in 16 patients treated with SMS 201-995 infusion was 84ml/min (sem 9). The values for the two groups are not significantly different ($p=0.1$).

Chest infection developed after 17 of the 39 bleeds evaluable. The patient who died at 4.5 hours has not been included. The details of treatment and the incidence of uncontrolled or recurrent bleeding in these cases are shown in Table 4. There is a significant association between chest infections and rebleeding ($p=0.05$) but no association between chest infection and treatment with oesophageal tamponade ($p=0.25$).

**Table 4** Incidence of chest infections in comparison with type of treatment and occurrence of rebleeding

| | *Chest infection* | *Total number of patients in group* |
|---|---|---|
| SMS 201–995 infusion group | 7 | 20 |
| Oesopageal tamponade group | 10 | 19 |
| SMS 201–995 infusion alone | 4 | 14 |
| SMS 201–995 and tamponade | 4 | 7 |
| Oesophageal tamponade alone | 9 | 18 |
| Uncontrolled bleed or rebleed | 10 | 15 |
| No further bleed | 7 | 24 |

### *Survival*

None of the 20 bleeds in the SMS 201-995 group proved fatal, in comparison with 5 deaths from the 20 bleeds in the oesophageal tamponade group ($p=0.47$). Three patients died of hepatorenal failure at 4.5 hours, 40 hours and 9 days from entry to the study. One patient died at 9 days from entry to the study when he suddenly had a further haematemesis, aspirated and could not be resuscitated. The fifth patient died at four days from study entry after an oesophageal perforation had occurred at rigid oesophagoscopy. Rigid oesophagoscopy and sclerotherapy had been performed on two occasions within 48 hours in order to try to control the patient's variceal bleeding which had recurred immediately after fibreoptic endoscopy and sclerotherapy. This patient was not felt to be fit for any other intervention. All five patients who died were modified Child's grade C.

### DISCUSSION

In this trial a 25$\mu$g/hr intravenous infusion of SMS 201-995 controlled bleeding during the first four hours of treatment in 18 of 20 active bleeds from oesophageal varices. However the control rate had fallen to 70% by 24 hours and 50% by 48 hours. Oesophageal tamponade was able to control more bleeds without crossover, although this difference did not reach statistical significance in the relatively small number of bleeds studied.

Two previous controlled clinical trials have compared the naturally occurring tetradecapeptide somatostatin with vasopressin in variceal bleeding. The results of the present study are similar to the results of Kravetz *et al.*[9], where 16 of 30 episodes of variceal haemorrhage were controlled by somatostatin for 48 hours, although inferior to the results of the Liverpool group[10], who found that variceal haemorrhage was controlled for 24 hours by somatostatin infusion in all 10 patients in whom it was employed. Since we have shown a deterioration in control of bleeding with time, the difference in results may be due to the longer trial period in both the study by Kravetz and colleagues[9] and the present study. In the study of Jenkins and co-workers[10], somatostatin was only given to ten patients and the small numbers in all of the studies[9,10] may help account for the apparent differences. In more recent placebo controlled studies, one multicentre trial[14] failed to show significant benefit in that in the 30 hour study period 65% of the patients given somatostatin stopped bleeding as opposed to 83% of those given placebo. Despite the somewhat surprising "success" of placebo, transfusion requirements and mortality rates were similar in the two groups. In a trial from the Royal Free Hospital, London, somatostatin proved significantly more effective than placebo in controlling bleeding (64% versus 41%) and when bleeding recurred it did so earlier in the placebo group[15]. Somatostatin significantly reduced blood and plasma transfusion requirement and halved the need for balloon tamponade. Although it was concluded that somatostatin was safe and more effective at controlling bleeding than placebo, 30 day mortality was not affected.

In our situation we felt that a 48 hour trial comparing SMS 201-995 infusion with placebo would be difficult to justify and we have therefore compared SMS 201-995 infusion with the previous best means of immediate treatment in our department[2]. The fact that SMS 201-995 infusion was carried out for 48 hours is balanced by the fact that the Minnesota tube was left in place for this period so that tamponade could be continued if necessary. Although emergency sclerotherapy may be the ideal means of treatment the necessary expertise and facilities are not always immediately available and some means of emergency treatment is essential. Previous controlled trials in variceal bleeding have achieved variable control rates with placebo — ranging from 25% at 24 hours[16], through 37% at 8 hours[17] to 45% at 24 hours[18]. Strict comparison of trials is difficult because of differences in the severity of bleeds included, in the accuracy of diagnosis, in the means of conservative treatment, in the length of the trial period and in the methods used to assess control of haemorrhage. Furthermore, although the number of bleeding episodes in the current trial is similar to other recent controlled trials[9,10], it must not be forgotten that the number of variceal bleeds required in a controlled trial to achieve even 50% power, assuming a substantial difference in bleeding control rates, is more than two hundred.

Although oesophageal tamponade has proved an effective means of controlling variceal bleeding in the past, it has several potential serious side-effects and a comparison of the incidence of side-effects in the two groups in this trial is of considerable importance. In the present study discomfort from the Minnesota tube was a major complaint in 22 of the 26 patients treated with tamponade and this dislike of tamponade contrasted markedly with the lack of symptomatic side-effects in the patients treated only with SMS 201-995 infusion. Despite the fact that somatostatin plays a role in blocking both insulin and glucagon release at physiological levels (19) only one patient developed an abnormality of glucose homeostasis during SMS 210-995 infusion and this was controlled easily. Vora *et al.*[20] have reported a reduction in renal function due to SMS 201-995. In patients with portal hypertension and actively bleeding varices many factors can influence a relatively simple measurement of renal function such as creatinine clearance but there was no significant difference in creatinine clearance rates in the two groups of patients in the present study. Furthermore no patient posed clinical problems with renal function while receiving an infusion of SMS 210–995.

Previous reports regarding the use of oesophageal tamponade have emphasised the risk of chest complications with this treatment. Novis *et al.*[21] noted 8 chest infections in 40 bleeds while Conn *et al.*[22] reported 8 cases of aspiration and one case of respiratory obstruction in 40 bleeds. In a complex clinical situation the exact cause of such chest infections is difficult to assess. However, if this incidence of chest infection was associated with tamponade alone, a reduction in this complication would have been expected in the group of patients treated with SMS 201-995 infusion only. This study has shown (Table 4) that chest infection was associated significantly with rebleeding or continued bleeding but not with tamponade. The finding that oesophageal tamponade was not associated with an increased risk of chest infection is initially surprising in view of the previous reports of this complication[1,21,22,23]. However in these severely ill patients there are a number of factors such as vomiting, sedation and endoscopy which might increase the risk of chest infection. It is difficult to determine which factor is of greatest importance.

In comparing treatments for variceal haemorrhage it is also worth considering that the administration of a drug infusion requires less expertise on the part of the medical and nursing staff than oesophageal tamponade. This may be a significant advantage in hospitals where relatively few patients with variceal bleeding are seen and an SMS 201-995 infusion may facilitate the patient's safe transfer to a specialised centre. The reduction in bleeding control rate with time emphasises that immediate treatment of variceal haemorrhage to control bleeding should be followed rapidly by definitive treatment to prevent rebleeding such as injection sclerotherapy. It is clear in retrospect that this definitive treatment should be performed earlier than 48 hours.

In this trial five patients were excluded because they had been judged to be exsanguinating by the staff of the Accident and Emergency Department and had been treated immediately by oesophageal tamponade, the standard hospital treatment. SMS 201-995 was not available to the A&E staff at that time. This may have excluded some of the severe bleeds from this trial but our strict entry criteria of active variceal bleeding at endoscopy probably also excluded a number of mild variceal bleeds. It may be that oesophageal tamponade will be necessary for the most severe bleeds, although its use was progmatic in the cases described above.

The main aim of this trial was to assess the effect of SMS 201-995 on bleeding from oesophageal varices and the finding of a higher survival rate in the SMS 201-995 group was unexpected. Stratification to account for liver function was not practical in a small trial and despite randomisation there was a greater number of patients with poor liver function in the tamponade group. Therefore the difference in survival may be artefactual due to the small numbers.

In conclusion, infusion of SMS 201-995 would appear to have certain advantages over oesophageal tamponade for the immediate control of bleeding oesophageal varices, with any reduction in bleeding control being offset by its better tolerability. However, control deteriorates with time and it is possible that its future role may be in the temporary control of bleeding while the patient is resuscitated before acute injection sclerotherapy is undertaken.

### *Acknowledgements*

We are grateful to Sandoz for supplying SMS 201-995 for this study and to the nursing and medical staff of the Royal Infirmary, Glasgow, for their help in the clinical care of these patients.

### *References*

1. Panes, J., Teres, J., Bosch, J. and Rodes, J. (1988) Efficacy of balloon tamponade in treatment of bleeding gastric and oesophageal varices. *Dig. Dis. Sci.*, **33**, 454–459
2. Haddock, G., Garden, O.J., McKee, R.F., Anderson, J.R. and Carter, D.C. (1989) Eosphageal tamponade in the management of acute variceal hemorrhage. *Dig. Dis. Sci.*, **34**, 913–918
3. Pitcher, J.L. (1971) Safety and effectiveness of the modified Sengstaken-Blakemore tube: a prospective study. *Gastroenterol.*, **61**, 291–298
4. Fogel, M.R., Knauer, M., Andres, L.L., Anmol, M. *et al.* (1982) Continuous intravenous vasopressin in active upper gastrointestinal bleeding. *Ann. Intern. Med.*, **96**, 565–569
5. Gimson, A.E.S., Westaby, D., Hegarty, J. (1986) A randomised trial of vasopressin plus nitroglycerin in the control of acute variceal haemorrhage. *Hepatology*, **6**, 410–413
6. Tsai, Y.T., Lay, C-S., Lai, K-H. (1986) Controlled trial of vasopressin plus nitroglycerin versus vasopressin alone in the treatment of bleeding oesophageal varices. *Hepatology*, **6**, 406–409
7. Westaby, D., Hayes, P.C., Gimson, A.E.S., Polson, R. and Williams, R. (1986) Injection sclerotherapy for active variceal bleeding: a controlled study. *Gut*, **27**, A1246
8. Pringle, S.D., McKee, R.F., Garden, O.J., Lorimer, A.R. and Carter, D.C. (1988) The effect of SMS 201-995 on portal and systemic haemodynamics in cirrhosis. *Alimentary Pharmacol. Ther.*, **2**, 451–459
9. Kravetz, D., Bosch, J., Teres, J., Bruix, J., Rimola, A., Rodes, J. (1984) Comparison of somatostatin and vasopressin infusions in treatment of acute variceal haemorrhage. *Hepatology*, **4**, 442–446
10. Jenkins, S.A., Baxter, J.N., Corbett, W., Devitt, P., Ware, J. and Shields, R. (1985) A prospective randomised controlled clinical trial comparing somatostatin and vasopressin in controlling acute variceal haemorrhage. *Br. Med. J.*, **i**, 275–278
11. Mulvihill, S., Pappas, T.N., Passaro, E. and Debas, H.T. (1986) The use of somatostatin and its analogues in the treatment of surgical disorders. *Surgery*, **100**, 467–475
12. Bauer, W., Briner, U., Doepfner, W. *et al.* (1982) SMS 201-995: a very potent and selective octapeptide analogue of somatostatin with prolonged action. *Life Sci.*, **31**, 1133–1140
13. Pugh, R.N.H., Murray-Lyon, I.M., Dawson, J.L. Pietroni, M.C. and Williams, R. (1973) Transection of the oesophagus for bleeding oesophageal varices. *Br. J. Surg.*, **60**, 646–649
14. Valenzuela, J.E., Schubert, T., Fogel, M.R. *et al.* (1989) A multicenter, randomised double-blind trial of somatostatin in the management of acute haemorrhage from esophageal varices. *Hepatology*, **10**, 958–961
15. Burroughs, A.K., McCormick, A., Hughes, M.D., Sprengers, D., D'Heygere, F. and McIntyre, N. (1990) Randomised, double blind placebo controlled trial of somatostatin for variceal bleeding. *Gastroenterology*, **99**, 1388–1395
16. Conn, H.O., Ramsby, G.R., Storer, E.H. *et al.* (1975) Intra arterial vasopressin in the treatment of upper gastrointestinal hemorrhage; a prospective, controlled clinical trial. *Gastroenterol.*, **68**, 211–221

17. MacDougall, B.R.D., Bailey, R.J. and Williams, R. (1977) H2 receptor antagonists and antacids in the prevention of acute gastrointestinal haemorrhage in fulminant hepatic failure. *Lancet*, **i**, 617–619
18. Merigan, T.C., Poltkin, G.R. and Davidson, C.S. (1962) Effect of intravenously injected posterior pituitary extract on haemorrhage from bleeding oesophageal varices. *N. Engl. J. Med.*, **266**, 134–135
19. Bloom, S.R. (1978) Somatostatin and the gut. *Gastroenterol.*, **75**, 145–147
20. Vora, J.P., Owens, D.R., Ryder, R., Atiea, J., Luzio, S. and Hayes, T.M. (1986) Effect of somatostatin on renal function. *Br. Med. J.*, **i**, 1701–1702
21. Novis, B.H., Duys, P., Barbezat, G.O., Clain, J., Bank, S. and Terblanche, J. (1976) Fibreoptic endoscopy and the use of the sengstaken tube in acute gastrointestinal haemorrhage in patients with portal hypertension and varices. *Gut*, **17**, 258–263
22. Conn, H.O. and Simpson, J.A. (1967) Excessive mortality associated with balloon tamponade of bleeding varices. *JAMA*, **202**, 587–591
23. Read, A.E., Dawson, A.M., Kerr, D.N.S. and Turner, M.D. (1960) Bleeding oesophageal varices treated by oesophageal compression tube. *Br. Med. J.*, **i**, 227–231

*(Accepted by S. Bengmark, 13 January 1992)*

